# Medical Association of Georgia—1887

**Published:** 1887-05

**Authors:** 


### MEDICAL ASSOCIATION OF GEORGIA—1887.

The Association met in the Senate Chamber in Atlanta on the 20th of April, and was called to order by the President, Dr. T. O. Powell. Prayer was offered by Rev. Dr. Morrison; an appropriate and cordial address of welcome was then made by Dr. J. F. Alexander, which was well responded to by Dr. Eugene Foster, of Augusta. Our space will not permit of an extended notice of the proceedings.

In his address to the body, Dr. T. O. Powell, the President, very properly departed from the usual stereotyped remarks upon the duties of medical societies, and discussed a subject of great interest both to the profession and the public at large. His theme was Heredity and Environment—the transmission of both Moral and Physical Peculiarities from Parent to Child—and the great importance of the fact was forcibly and ably presented. The physician should bear in mind this truth, not only in its professional bearings but should impress the public with its importance. Healthful families should not marry into defective or diseased families. He had found, in his observations upon the insane, that a majority of insane cases were from hereditary transmission. It was the province of the physician to inform the people of the dangers of hereditary transmission, that they may guard against them. Marriages of healthful with defective constitutions should be avoided. The stock-breeders have improved, and developed fine breeds by observing the law of heredity ; the principle will hold good with respect to human beings. The fact of heredity, especially the transmission of vices, should be taught in our schools. It would be well if there was a board of health in every county to say if a marriage license should be granted. Many of the offspring of improper marriages find their way into our penetentiaries. We have compulsory laws in regard to small-pox, vaccination, etc.; why not in relation to marriage also ? Families with neuralgic, insane, alcoholic, and syphilitic diatheses should not intermarry. The natural or hereditary tendencies of children should be noted and utilized. Dr. Powell referred to the physical and moral defects of immigrants as tainting our population and increasing crime and pauperism in our country, and gave statistics in proof of this. He favors the establishment by the State of schools for the feeble minded, a hospital for the insane, and a house of correction. We regret that we have not space for further notice of this able and interesting address.

Many interesting subjects were discussed by the Association—among them the subject of Expert Testimony ; the bill before the Legislature on Practical Anatomy, and the Alabama Medical Organization. On the latter subject Dr. Cochran, the great organizer of Alabama, was introduced by Dr. Baird and made an interesting address. The report in reference to the Alabama plan of organization was, under the constitution, laid over until next session, when it must come up for final action.

A resolution sympathizing with the present efforts to make successful the International Medical Congress elicited considerable discussion, but was finally adopted.

The generous hospitality extended to the Association by the physicians and citizens of Atlanta evoked many expressions of appreciation and gratitude. The excursion to Salt Spring, and the elegant feast given by our generous fellow-citizen Mr. E. W. Marsh, the proprietor of this celebrated health resort, was one of the most enjoyable occasions we have ever participated in. The members of the Association were delighted with Salt Springs, with the water, the attractions and surroundings of the locality, the mangnificent hotel in process of construction, and with the great advantages of the place as a health resort. An address of thanks, in behalf of the Association was made by one of its members and responded to by Col. Howell of the Constitution. Mr. Marsh and Mr. Inman were present on the occasion and extended personally and cordially that generous hospitality for which high-bred and intelligent Georgians are noted.

The entertainment at the Ivy Street Hospital, the visit to the Southern Medical College, the Governor's reception, and the great banquet at the Kimball House were all most happy and enjoyable occasions, and will long be remembered by the large assemblage of medical men constituting the Medical Association of Georgia.

The officers elected for the ensuing year are :

President—Dr. A. G. Whitehead, of Waynesborough.

Vice-Presidents—Dr. A. Smith, Hawkinsville ; Dr. John Gerdine, Athens.

Secretary—Dr. James A. Gray, Atlanta.

Treasurer—Dr. E. C. Goodrich, Augusta.

Censor—Dr. M. H. O'Daniel, Milledgeville.

The appointments of Chairmen for Sections Reports are as follows :

First District—Practice, Dr. J. B. Read ; Surgery, Dr. J. D. Martin ; Gynæcology, Dr. R. J. Nunn.

Second District—Practice, Dr. B. R. Doster ; Surgery, Dr. P. S. Hillsman ; Gynæcology, Dr. J. A. Chappell.

Third District—Practice, Dr. J. A. Fort ; Surgery, Dr. N. P. Jelks ; Gynæcology, Dr. R. G. Engram.

Fourth District—Practice, Dr. J. F. Cole ; Surgery, Dr. J. W. Griggs, Gynæcology, Dr. A. W. Griggs.

Fifth District—Practice, Dr J. B. Baird ; Surgery, Dr. W. S. Elkin ; Gynæcology, Dr. W. H. Poole.

Sixth District—Practice, Dr. W. F. Holt ; Surgery, Dr. I. L. Harris ; Gynæcology, Dr. J. Emmett Blackshear.

Seventh District—Practice, Dr. W. G. England ; Surgery, Dr. F. R. Calhoun ; Gynæcology, Dr. Robert Battey.

Eighth District—Practice, Dr. A. S. J. Stone ; Surgery, Dr. Samuel C. Benedict ; Gynæcology, Dr. M. P. Deadwyler.

Ninth District—Practice, Dr. W. A. Watson ; Surgery, Dr. L. G. Hardeman ; Gynæcology, Dr. J. W. Bailey.

Tenth District—Practice, Dr. A. C. Davidson ; Surgery, Dr. DeSaussure Ford ; Gynæcology, Dr. H. F. Campbell.

---

THE J. P. BUSH MANUFACTURING CO. was represented at the Georgia Medical Association by a very pleasant and attractive lady agent, Mrs. Vivian. Their Bovinine preparation is a good one.

PARKE, DAVIS & CO. had on exhibition at the Georgia Medical Association a very large and varied supply of drugs, chemicals, etc. It was quite refreshing to the village and country physicians, coming up from remote sections of the State, to look at their beautiful preparations, especially their normal liquids, sugar coated pills and elastic capsules, coca and cascara cordial, etc. Dr. Stone, their gentlemanly and accommodating agent, was present to explain and show up everything—in which duty he cannot well be excelled. He also had a photographic apparatus and took a negative of the entire body of the members of the Association, and will present each member a picture of the group. The members were very much pleased with this performance, which was attributed to the kindness and partiality of Messrs. Parke, Davis & Co. for Doctors, and to the fact that they were all men of fine *physique* (physic !)

R. A. ROBINSON & CO.—This excellent firm made a beautiful display of their preparations at the Georgia Medical Association. Their hypophosphite ; lime juice and pepsin ; phosphoric elixir ; wine of coca are all good preparations, containing fine medicinal agents well combined and neatly and elegantly prepared. See the advertisement of this house in this journal.

BUDWELL'S EMULSION OF COD-LIVER OIL.—We invite attention to the advertisement of Budwell's Emulsion of Norwegian Cod-Liver Oil combined with the iodides of arsenic, calcium and manganese. We regard this as an admirable combination. The excellent house of Budwell & Christian made an exhibit of samples of their goods at the recent meeting of the Georgia Medical Association. We were much pleased with the exhibit and the polite gentleman in charge and shall take occasion to refer to their preparations again.

DOLIBER, GOODALE & CO. were well represented at the Georgia Medical Association. Their Mellin's Food is a fine preparation for children.

THE WM. S. MERRELL CHEMICAL CO., of Cincinnati, had also a beautiful display. Also, JOHN REYNDUS & CO.; the GALVANO-FARDIC CO. of New York, and JOHN WYETH & BROTHERS. Of these we expect to make more special mention when we receive their advertisements for the RECORD, which we hope soon to do.

